# The Diverse Roles of Histone Demethylase KDM4B in Normal and Cancer Development and Progression

**DOI:** 10.3389/fcell.2021.790129

**Published:** 2022-02-02

**Authors:** Zhongze Wang, Huarui Cai, Erhu Zhao, Hongjuan Cui

**Affiliations:** ^1^ State Key Laboratory of Silkworm Genome Biology, Medical Research Institute, Southwest University, Chongqing, China; ^2^ Engineering Research Center for Cancer Biomedical and Translational Medicine, Southwest University, Chongqing, China

**Keywords:** KDM4B, histone demethylase, therapeutic target, cancer treatment, KDM4 inhibitor

## Abstract

Histone methylation status is an important process associated with cell growth, survival, differentiation and gene expression in human diseases. As a member of the KDM4 family, KDM4B specifically targets H1.4K26, H3K9, H3K36, and H4K20, which affects both histone methylation and gene expression. Therefore, KDM4B is often regarded as a key intermediate protein in cellular pathways that plays an important role in growth and development as well as organ differentiation. However, KDM4B is broadly defined as an oncoprotein that plays key roles in processes related to tumorigenesis, including cell proliferation, cell survival, metastasis and so on. In this review, we discuss the diverse roles of KDM4B in contributing to cancer progression and normal developmental processes. Furthermore, we focus on recent studies highlighting the oncogenic functions of KDM4B in various kinds of cancers, which may be a novel therapeutic target for cancer treatment. We also provide a relatively complete report of the progress of research related to KDM4B inhibitors and discuss their potential as therapeutic agents for overcoming cancer.

## 1 Introduction

**FIGURE 1:**
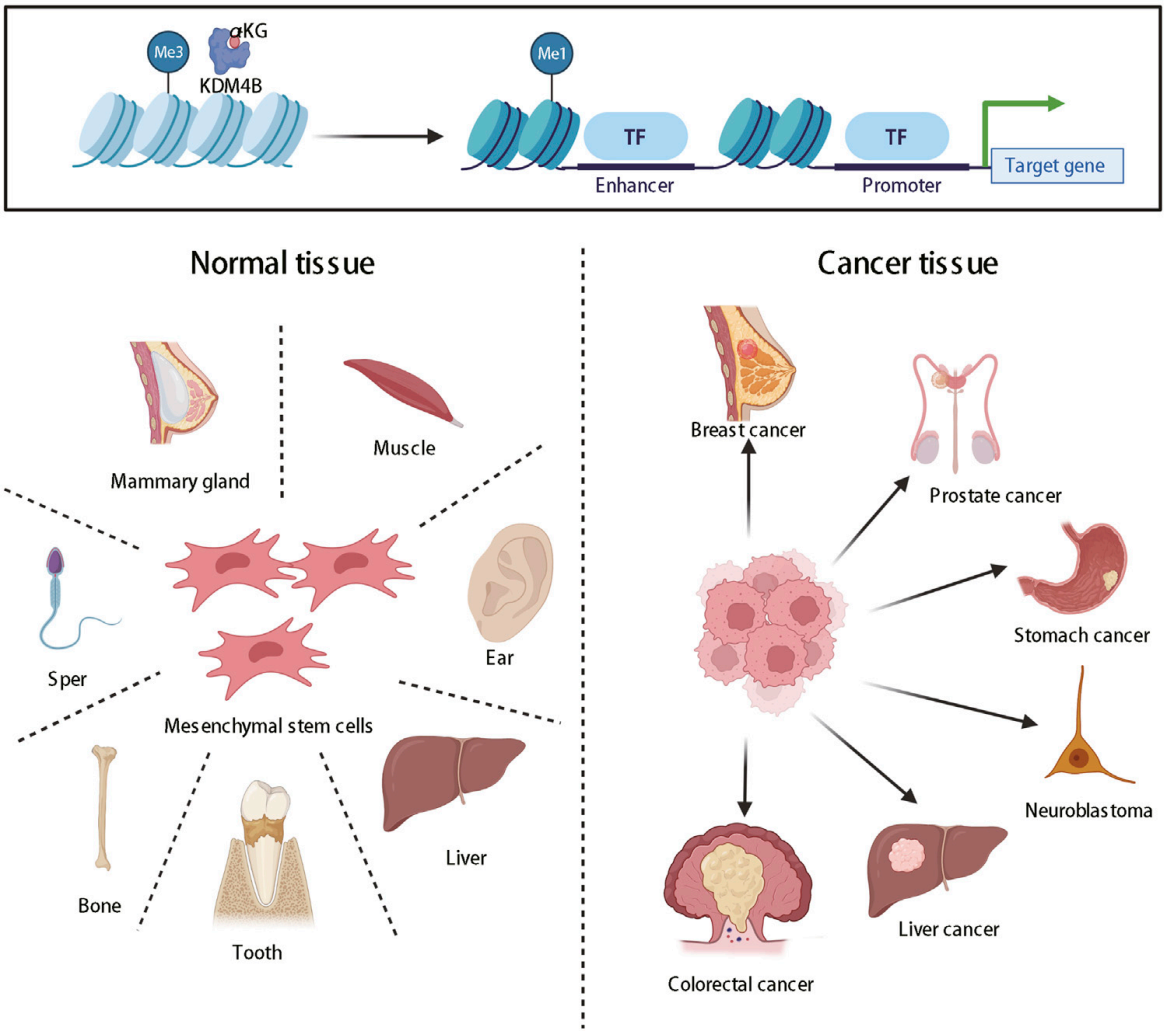
The roles of KDM4B in organ differentiation and tumor formation. KDM4B switches on gene expression through histone demethylation and produces pleiotropy, which can promote not only the normal differentiation of most organs but also tumor formation.

Histone methylation states were uniformly regarded as static and unchangeable until the discovery of histone lysine demethylases, thereby changing this ideology, and partial histone methylation states are now considered as dynamic reversible processes (Shi and Whetstine, 2007). KDM4 (JMJD2) family proteins, which are generally located in the nucleus, are a class of histone lysine demethylases that remove methyl groups from lysine residues in histone tails, thereby regulating the transcriptional activity of target genes (Chen et al., 2006). KDM4 family includes six demethylases named KDM4A-F. KDM4A-D are highly expressed in cardiovascular diseases, mental retardation, and multiple cancers, including prostatic cancer, breast cancer, neuroblastoma, and so on, and thus have emerged as potential broad-spectrum therapeutic targets (Young and Hendzel, 2013; Zhao et al., 2016; Lee et al., 2020). KDM4E is an epigenetic regulator for embryonic development and differentiation (Liu et al., 2018). However, the roles of KDM4E and 4F in cancers have rarely been reported. In this review, we mainly focus on the KDM4B protein of the KDM4 family. KDM4B mainly turns on the expression of genes through histone demethylation, which can regulate stem cells self-renewal and differentiation (Mak et al., 2021) and the development of a variety of organs (Guo et al., 2011; Jun et al., 2011; Uribe et al., 2015). At present, KDM4B has been proved to induce the differentiation of mesenchymal stem cells, which has significant physiological significance for sexual organ formation and development (Kawazu et al., 2011), bone formation (Lee et al., 2016), liver development (Guo et al., 2011), and invagination of inner ear (Uribe et al., 2015) (Figure 1). However, its abnormal expression is closely related to the occurrence and development of human diseases, such as cancers (Berry and Janknecht, 2013) (Figure 1).

In recent years, inhibitors of KDM4B have been developed, providing more new ideas for targeted cancer therapy. However, we have to face the challenge that KDM4B is a very essential gene in human, deletion of which could result in unwanted side effects. For example, KDM4B deletion gene in mice has been reported to cause osteoporosis (Deng et al., 2021) and severe obesity (Kang et al., 2018). Meanwhile, KDM4B is crucial for DNA damage repair (Castellini et al., 2017). Although the loss of KDM4B will enhance the effect of radiotherapy, the rate of DNA error in normal tissues will also increase, which will cause unpredictable consequence (Hsieh et al., 2018; Xiang K. et al., 2020). Therefore, KDM4B as a cancer target must be supported by more animal and clinical data, and new cancer treatments need to be developed and used.

## 1.1 Structure and Function of KDM4B

The human KDM4B gene is located in band 13 of the long arm of chromosome 19 (19p13.3), and the gene sequence length is approximately 184.5 kb. After splicing, three mRNAs with three different lengths of 5.6, 3.1 and 1.1 kb are generated and can be translated into three proteins with sizes of 1,096, 448, and 429 aa. Only the first protein, which is called KDM4B, contains complete JmjN, JmjC, PHD, and Tudor domains, with a protein size of approximately 121.9 kDa, while the latter two do not have the full functionality of KDM4B because they contain only JmjN and JmjC domains. The JmjC domain, the principal region driving histone demethylation functions, contains an Fe (2+) active center site (Chen et al., 2006), which uses α-ketoglutarate and O_2_ as substrate cofactors to remove trimethyl or dimethyl moieties on histone terminal lysine by hydroxylation reactions (Cloos et al., 2006). However, cobalt ions can inhibit histone demethylase activity and induce histone modification changes by competing with ferric ions for binding to the active center of the JmjC domain (Li et al., 2009). The JmjN domain is usually next to the JmjC domain and can physically interact with the JmjC domain via two β-sheets to help maintain the demethylase activity of the JmjC domain (Shin and Janknecht, 2007b; Quan et al., 2011). In the KDM4A/C proteins, the JmjN domain can mediate homodimerization or heterodimerization, which is critical for their demethylase activity (Levin et al., 2018). Many studies have found that PHD domain and Tudor domain can specifically bind to corresponding histone trimethylation sites to help the accurate localization of enzyme action (Shin and Janknecht, 2007b; Iwase et al., 2007; Xiang Y. et al., 2020). Compared to other histone demethylases in the family, KDM4B has a weak demethylation ability, mainly catalyzing the demethylation of H3K9me3, H3K9me2, H3K36me3, H3K36me2, H1.4K26me3, and H4K20me3 (Fodor et al., 2006; Trojer et al., 2009; Chu et al., 2014; Su et al., 2016; Xiang Y. et al., 2020).

## 2 The Roles of KDM4B in Normal Development

### 2.1 *KDM4B and Stem Cell Development*

In human embryonic stem cells (hESCs), KDM4B expression is necessary for ESC self-renewal and the generation of induced pluripotent stem cells (Das et al., 2014), suggesting that sustained removal of H3K9 methylation near promoters by KDM4 demethylases is essential for ESC self-renewal and early development (Pedersen et al., 2016). In ESCs, there is an interesting phenomenon in which KDM4B and Nanog control each other through a regulatory loop. Through a demethylation reaction, KDM4B not only switches on the transcription of Nanog, a key gene of ESCs, but also participates in the transcriptional regulation of downstream genes of Nanog, while the expression of KDM4B is also regulated by Nanog (Das et al., 2014).

Artificially induced overexpression of KDM4B in ESCs significantly reduces the overall chromatin H3K9me3 content and increases the survival of cloned embryos *in vitro* by 30% (Antony et al., 2013). In addition, KDM4 family proteins have been found to bind JAK2 and thereby maintain the undifferentiated state of mouse ESCs (mESCs) (Wulansari et al., 2021). This finding suggests that the presence of KDM4-related proteins in ESCs may inhibit cell differentiation and promote cell proliferation. Recent studies have demonstrated that KDM4B regulates and maintains gene transcription in the form of the KDM4B-TFAP2C-LSD1 complex in human trophoblast stem cells, thus protecting the normal development of trophoblast cells (Mak et al., 2021).

KDM4B has also been reported to be involved in and to enhance the inflammatory response of neural stem cells; knockdown of KDM4B in neural stem cells results in increased H3K9me3 at the promoters of the Notch1, IL-1 and IL-2 genes, thereby inhibiting the expression of the genes (Das et al., 2013). In addition, normal KDM4B expression in hematopoietic stem cells is required for the long-term maintenance of hematopoiesis (Agger et al., 2019).

### 2.2 *KDM4B and Reproductive System Development*

Downregulation of H3K9me3 is necessary for spermiogenesis, which is mainly regulated by KDM4B and KDM4D (Iwamori et al., 2011). During spermatogenesis, the key gene KDM4B is upregulated early and rapidly to reduce the level of H3K9me3 of multiple differentiated genes and to activate their expression, thereby promoting sperm maturation (Yoshioka et al., 2009). However, KDM4D is the main histone demethylase during spermatogenesis, and KDM4B plays a secondary role (Iwamori et al., 2011).

In the mammary gland, KDM4B acts as a cofactor of estrogen receptor (ER) to promote mammary gland development and maturation (Kawazu et al., 2011). The transcription factor ERα promotes the expression of KDM4B, and recruits it to activate the transcription of MYB, MYC, and CCND1 though histone demethylation (Kawazu et al., 2011).

### 2.3 *KDM4B and Adipose Tissue Differentiation*

KDM4B plays an important role in adipocyte differentiation of 3T3-L1 adipocyte precursors. As a histone demethylase, KDM4B reduces H3K9me3/me2 content in the peroxisome proliferator-activated receptor (PPAR) and CAAT/enhancer binding protein (C/EBP) promoters, in turn stimulating the expression of PPAR and C/EBPα as well as activating adipogenesis (Jang et al., 2017). In addition, C/EBPβ have been found to bind to the KDM4B promoter to activate the expression of KDM4B (Guo et al., 2012). Moreover, KDM4B interacts with C/EBP β, is recruited to C/EBP β -regulated cell cycle gene promoters, including the Cdc45I, Mcm3, Gins1 and Cdc25c promoters, and it demethylates H3K9me3 residues and activates transcription, ultimately promoting adipocyte differentiation (Guo et al., 2012). However, in mature adipocytes, KDM4B in turn acts to enhance metabolism, thereby counteracting obesity (Cheng et al., 2018; Kang et al., 2018). KDM4B directly controls the expression of several metabolic genes, including Ppargc1a and PPARα (Cheng et al., 2018). Loss of KDM4B in mice results in increased fat mass and increased expression levels of lipogenesis-related genes, thereby leading to obesity associated with reduced energy expenditure and impaired adaptive thermogenesis, which is often accompanied by hyperlipidemia, insulin resistance, and liver and pancreas pathology (Cheng et al., 2018; Kang et al., 2018).

### 2.4 *KDM4B and Bone Formation*

KDM4B mainly induces osteoblastic differentiation of human mesenchymal stem cells (Ye et al., 2012; Qu et al., 2014; Lee et al., 2016; Yang et al., 2019; Liu et al., 2020). TGF-β significantly induces the expression of KDM4B in human mesenchymal cells (Lee et al., 2016). Overexpressed KDM4B is recruited to the SOX9 promoter to eliminate the H3K9me3 marker, thus cooperating with SMAD3 to activate SOX9 expression, which ultimately promotes chondrogenic differentiation of mesenchymal stem cells (Lee et al., 2016). Ascorbate contributes to chondrogenesis by promoting the expression of KDM4B, which upregulates chondrogenic differentiation genes, including CD44, CD73, CD105, and Runx2 (Liu et al., 2020).

In the osteogenic induction of dental tissue-derived mesenchymal stem cells, KDM4B, Distal less homeobox 2 (DLX2) and Distal less homeobox 5 (DLX5) are first upregulated by BMP4 (Qu et al., 2014; Yang et al., 2019). Then, DLX5 can enhance the transcription of KDM4B by directly binding to the KDM4B promoter (Yang et al., 2019). KDM4B also upregulates the expression of DLX2 and DLX5 by removing H3K9me3 markers (Ye et al., 2012; Qu et al., 2014; Yang et al., 2019), thus producing high expression of DLX5 and DLX2 through a positive feedback regulatory mechanism. These proteins not only enhance the activation of alkaline phosphatase in cells and the ability of cells to mineralize but also increase the expression of dentin sialophosphoprotein (DSPP), dentin matrix acidic phosphoprotein 1 (DMP1), osteopontin (OPN), and osterix (OSX), thereby promoting tooth formation (Qu et al., 2014; Yang et al., 2019).

Human vascular smooth muscle cells can also be transformed into osteoblast-like cells (Kurozumi et al., 2019). In this process, KDM4B is recruited by IL-6/sIL-6R to induce demethylation of H3K9me3 markers in the STAT3-binding site in the Runx2 promoter region, thus allowing STAT3 to bind to this site to turn on Runx2 transcription and ultimately induce the onset of transformation (Kurozumi et al., 2019).

During osteoclastogenesis, KDM4B physically associates with ccar1-med1 to form a complex that localizes to the promoters of multiple osteoclast-related genes and it promotes the expression of related genes by altering the chromatin structure near the promoters through the function of histone demethylases (Yi et al., 2021). KDM4B is a key regulator of osteoclast formation, and inhibition of KDM4B protein in mouse experiments prevents bone loss but creates osteoporotic conditions (Yi et al., 2021). Upon knockdown of KDM4B, the osteogenic differentiation capacity of mesenchymal stem cells is diminished, but adipogenic differentiation is enhanced (Ye et al., 2012). Loss of KDM4B increases global H3K9me3, thereby reducing bone formation and increasing marrow fat to exacerbate skeletal aging and osteoporosis (Deng et al., 2021).

### 2.5 *KDM4B and Formation of Organs*

Several experiments have shown that KDM4B plays a key role in the development of the central nervous system (Das et al., 2013; Fujiwara et al., 2016), liver (Guo et al., 2011; Jun et al., 2011), ear (Uribe et al., 2015), and muscle (Choi et al., 2015). During neurogenesis of neural stem cells, KDM4B demethylates H3K9me3 markers in promoters of the Notch1, IL-1, and IL-2 genes. After KDM4B knockout, the expression of Notch1, IL-1, IL-2, P65, iNOS, Bcl2, and TGF-β is significantly inhibited (Das et al., 2013). KDM4B knockout mice show hippocampal abnormalities and exhibit hyperactivity, working memory deficits, and spontaneous seizure-like behavior (Fujiwara et al., 2016). In the developing liver of mice, the protein level of KDM4B in 2-week-old mice and 4-week-old mice is significantly higher than that in 6-week-old mice, indicating that the expression level of KDM4B plays a key role in the development of mouse liver; however, the expression level in mature liver cells was decreased (Guo et al., 2011). During ear development, KDM4B can demethylate H3K9me3 markers and directly regulate DLX3 expression, thus inducing inner ear invagination (Uribe et al., 2015). During myogenic differentiation of myoblasts, the expression of KDM4B is gradually increased and can be promoted by demethylation of H3K9me3 markers in promoters of myogenic regulators (MyoD and Myogenin), which ultimately promotes the generation of muscle cells (Choi et al., 2015).

## 3 The Roles of KDM4B in Various Kinds of Cancers

### 3.1 *Prostate cancer*

The control of KDM4B expression in prostate cancer tissue is abnormal. Compared to normal prostate tissue, the KDM4B protein level in prostate cancer tissue is significantly downregulated (Vieira et al., 2014). However, the viability of prostate cancer cells significantly decreases after KDM4B was blocked by drugs or gene knockout (Chu et al., 2014). Histone demethylases are mostly associated with stable genes such as genes involved in DNA repair, DNA recombination and chromosome organization, while the broad inhibitory nature of inhibitors and the low enzymes levels render prostate cancer sensitive to the loss of KDM4B.

KDM4B is involved in resisting androgen deprivation and promoting the development of prostate cancer into castration-resistant prostate cancer (Duan et al., 2019b; Shat et al., 2019). KDM4B overexpression leads to an increase in AR-V7, which is an alternative splice variant of the androgen receptor (AR). Under androgen deprivation (ADT), protein kinase A (PKA) accumulates and phosphorylates serine 666 of KDM4B, thus activating its function (Duan et al., 2019b). First, the demethylase activity of KDM4B modulates heterochromatin and promotes AR transcription (Coffey et al., 2013). Subsequently, activated KDM4B binds to DNA and enriches splicing factors, such as SF3B3. Finally, KDM4B recruits splicing factors to splice in specific regions of chromatin, transforming the precursor RNA of AR into the precursor RNA of AR-V7. The AR-V7 protein is a type of androgen receptor variants that can function as an AR without the influence of ADT (Nakasato et al., 2020). After knockout of KDM4B, the rapid decrease in AR-V7 content inhibits the growth of prostate cancer cells and enhances the efficacy of enzalutamide (Duan et al., 2019b). Recent research has found that KDM4B is also a coactivator of AR and controls the expression of the MYC gene through epigenetic regulation of H3K9 markers (Tang et al., 2020). Knockout of KDM4B in advanced prostate cancer cells inhibits the expression of the c-MYC gene, leading to improved treatment with enzalutamide (Tang et al., 2020). In castration-resistant prostate cancer cells, KDM4B activated autophagy by regulating the Wnt/β-catenin signaling, and autophagy inhibition attenuated KDM4B-induced cell proliferation (Shat et al., 2019). These results provide new insights into the role of KDM4B in driving the development of castration-resistant prostate cancer, revealing KDM4B may serve as a target for the treatment of this disease. Surprisingly, trichostatin A (TSA), a classic histone deacetylase that acts on prostate cancer cells, has been found to downregulate the KDM4B protein, causing downregulation of cyclin B1 and upregulation of the caspase-3 and caspase-9 apoptotic proteins, thereby inhibiting the growth of prostate cancer cells and promoting their apoptosis (Zhu et al., 2012). These studies provide the foundation for further exploration of KDM4B inhibitors as therapeutic drugs for prostate cancer.

### 3.2 *Breast cancer*

KDM4B is highly expressed in estrogen receptor (ER) positive breast cancer and is a key regulatory gene (Yang et al., 2010; Kawazu et al., 2011). Studies have shown that in ER-positive breast cancer, ERα acts synergistically with glucocorticoid receptors (GRs) to directly promote the expression of the KDM4B gene (Yang et al., 2010; West et al., 2016). KDM4B and KDM3A can synergistically promote the expression of ERα, forming a positive feedback loop (Jones et al., 2019). KDM4B demethylates specific H3K9me3 markers upstream of the ER gene, and its binding to GATA-3 drives ER gene expression (Gaughan et al., 2013). In breast cancer cells, the deletion of KDM4B not only reduces the transcription of WEE1, CCND1, and CCNA1 (Yang et al., 2010), but also disrupts the estrogen-induced cell cycle G1-S phase transition (Shi et al., 2011), causing breast cancer cells to stall at the G2-M phase (Yang et al., 2010) or G1-S phase (Shi et al., 2011). We previously found that KDM4B is recruited to the ERα target site where it demethylates H3K9me3 markers and promotes the transcription of ER response genes including MYB, MYC, and CCND1 (Kawazu et al., 2011). In ER-positive breast cancer, the miR-491-5p, a tumor suppressor gene, directly targets the mRNA of KDM4B, and its overexpression inhibits breast cancer cell proliferation (Zeng et al., 2015). A novel KDM4 inhibitor, NCDM-32B, has shown some utility in the treatment of a subset of aggressive breast cancers, in which it functions by disrupting several key pathways driving cell proliferation and transformation in breast cancer through inhibition of KDM4B expression (Ye et al., 2015).

PI3K-AKT inhibitors are specifically used for the treatment of triple negative breast cancer, but resistance can develop in the absence of PTEN (Wang et al., 2018; Gris-Oliver et al., 2020). KDM4B plays a key role in drug resistance, and knockdown of KDM4B or use of KDM4B inhibitors stimulates activation of the unfolded protein response pathway to produce eIF2α. The eIF2α interacts with cytoplasmic components, which abrogates the resistance of PTEN-null triple-negative breast cancer cells to PI3K-AKT inhibitors (Wang et al., 2018). However, KDM4B promotes the binding of DNA to topoisomerase 2.thereby improving the sensitivity of breast cancer cells to anthracyclines (Seoane et al., 2019). Therefore, KDM4B inhibitors cannot be used in conjunction with anthracyclines to treat breast cancer.

### 3.3 *Gastric cancer*

The expression of the KDM4B is increased in human primary gastric cancer tissues (Li et al., 2011). In addition, KDM4B has been identified as a gene required for the proliferation and growth of gastric cancer cells (Li et al., 2011; Kim et al., 2012; Zhao et al., 2013), and as a key gene required for *Helicobacter pylori*-induced gastric carcinogenesis (Han et al., 2016). Loss of KDM4B in gastric cancer cells, not only inhibits cell proliferation and induces apoptosis (Li et al., 2011), but also inhibits metastasis *in vivo* (Zhao et al., 2013). In gastric cancer, metastasis is common and frequent, and KDM4B can promote the epithelial-mesenchymal transformation of cancer cells by physically binding to β-catenin (Zhao et al., 2013). KDM4B and β-catenin both bind to the promoter of the target gene vimentin, and induce local demethylation of H3K9 markers to increase transcription, thereby promoting gastric cancer metastasis (Zhao et al., 2013). Another mechanism is that KDM4B upregulates miR-125b expression and activates the Wnt signaling pathway, which promotes gastric cancer metastasis (Jing et al., 2019). The miR-491-5p acts as an inhibitor of KDM4B in clinical gastric cancer, and it can bind to the 3′UTR of KDM4B mRNA, inhibit the expression of KDM4B and play an anticancer role (Zhang et al., 2018).

### 3.4 *Neuroblastoma*

In neuroblastoma, N-MYC physically interacts with the PHD/Tudor domain of KDM4B and recruits KDM4B to the promoter of N-MYC target genes, thus regulating the N-MYC signaling pathway (Yang et al., 2015). Knockdown of KDM4B significantly inhibits the growth of neuroblastoma cells and neurite outgrowth, indicating that KDM4B is an important gene for maintaining the morphology of neuroblastoma cells (Yang et al., 2015). Ciclopirox has also been found to be an effective KDM4B inhibitor that inhibits neuroblastoma cell proliferation, but has little effect on normal nerve cells (Yang et al., 2017). This shows that ciclopirox may be a potential treatment for neuroblastoma.

### 3.5 *Colorectal cancer*

In multiple reports, KDM4B has been found to be overexpressed in colorectal cancer cells and tissues (Fu et al., 2012; Berry et al., 2014; Li et al., 2016; Li et al., 2020), which is associated with worse prognosis of colorectal cancer patients (Li et al., 2020). In colorectal cancer tumor masses, cells become hypoxic because of clumping, and production of the hypoxia inducible factor alpha (HIF-1α) is induced, which directly activates KDM4B transcription and translation (Fu et al., 2012). After DNA is damaged, the transcription factor cAMP response element binding protein (CREB), which is frequently associated with worse prognoses and relapse following radiotherapies, has been found to directly activate KDM4B expression by binding to the conserved region of promoter (Deng et al., 2018). Under doxorubicin treatment, p53 can directly regulate the transcription of KDM4B to deal with DNA damage by binding to a specific P53 binding site on the KDM4B promoter, and KDM4B induction decreases the transcription of p53 downstream tumor suppressor genes p21 and PIG3 (Castellini et al., 2017). Furthermore, ectopic expression of KDM4B enhances tumor growth *in vivo* (Castellini et al., 2017). These data may provide new insights into KDM4B as a potential epigenetic target for therapeutic intervention of cancers harboring WT p53. In colorectal cancer cells, the KDM4B protein was found to interact with TCF4, β-catenin and ERG1 (sequence-specific transcription factor ETs-related gene 1), thereby promoting downstream expression of Jun, MYC, Cyclin D1, and TCL (the small GTPase TC10-like) (Berry et al., 2014; Chen et al., 2021). KDM4B can also directly activate the expression of the HAX1 gene and promote mitochondrial apoptosis in colorectal cancer cells (Li et al., 2016). Recently, KDM4B was found to accelerate energy metabolism by interacting with TRAF6 to ubiquitinate AKT, thus activating the AKT response pathway and leading to the upregulation of GLUT1 expression. This faster conversion of glucose to energy is required to meet the needs of rapidly growing cancer cells (Li et al., 2020).

Knockdown of KDM4B in colorectal cancer cells results in cell cycle arrest at the G2-M phase transition with induction of apoptosis and senescence (Chen et al., 2014). It may be that KDM4B is related to DNA damage repair. Once the DNA damage cannot be recovered, the cell will stall in the cycle preparation stage (Chen et al., 2014; Deng et al., 2018). The silencing of KDM4B can also lead to cell apoptosis, which is related to the down-regulation of apoptosis inhibitor protein Bcl-2 (Sun et al., 2014). In in vivo experiments, the intratumoural injection of KDM4B siRNA has been shown to result in tumors shrinking by the inhibition of the DNA damage response (Chen et al., 2014; Deng et al., 2018). This suggests that radiation therapy combined with KDM4B knockdown could be an effective treatment strategy.

### 3.6 Liver cancer

KDM4B is significantly upregulated in liver neoplasms than normal tissues, whose expression is positively correlated with tumor grade and Ki67, and KDM4B may be a potential diagnostic marker for liver cancer (Lu et al., 2015). A related radiotherapy sensitizer, emodin, has been found to reduce the expression of KDM4B to enhance the effect of radiotherapy (Hwang et al., 2015). In general, the combined treatment of radiotherapy and KDM4B inhibitors may be an effective strategy for the treatment of liver cancer.

### 3.7 Other cancers

In addition to the above cancers, KDM4B also plays an important role in the development of some other human cancers. In pancreatic cancer, KDM4B has been shown to regulate ZEB1 expression during TGF-β -induced EMT, and silencing KDM4B can significantly inhibit the migration and invasion of pancreatic cancer cells and EMT (Li et al., 2015). In ovarian cancer, KDM4B is induced under hypoxia, and deletion of KDM4B increases H3K9me3 level at target gene promoters such as the LOXL2, LCN2, and PDGFB promoters, thereby inhibiting the invasion, migration and globulization of ovarian cancer cells. KDM4B is a major factor affecting the prognosis of ovarian cancer (Wilson et al., 2017). However, in endometrial cancer, KDM4B modulates AR activity to promote endometrial cancer progression (Qiu et al., 2015) and was identified as a hub gene for cancer development by bioinformatics analysis (Zhang and Wang, 2019). The mechanism underlying the effects of KDM4B on myeloma remains largely unknown. However, studies have shown that triptolide decreases the levels of H3K4me2, H3K9me2, and H3K36me2 and induces apoptosis in myeloma cells by altering the expression of KDM4B (Wen et al., 2012). KDM4B is upregulated in osteosarcoma compared with normal tissues and can promote fibroblast growth factor 2 (FGF2) upregulation, which promotes the proliferation, migration, and invasion of osteosarcoma cells (Li and Dong, 2015). Although the significance of KDM4B in lung cancer remains to be elucidated, a large proportion of KDM4B-positive patients have significantly poorer prognosis than do KDM4B-negative patients, and KDM4B may serve as a new prognostic factor after lung cancer resection (Toyokawa et al., 2016). Functional inhibition of KDM4B reduces cisplatin resistance of non-small cell lung cancer (NSCLC) during the treatment of NSCLC (Duan et al., 2019a). The mechanism of KDM4B in leukemia is not well defined, and KDM4B expression is downregulated in chronic lymphoid leukemia (CLL) (Filiu-Braga et al., 2019), but KDM4B is known as an oncogene in acute lymphoid leukemia and its expression can be reduced using the spermidine analog Analogue-N 4-erucoyl Spermidine (Filiu-Braga et al., 2019). In classical Hodgkin lymphoma, high expression of KDM4B is significantly associated with worse prognosis and resistance to radiotherapy in patients (Bur et al., 2016).

Taken together, these findings clearly show that KDM4B acts as an oncogene in these other cancers. However, the carcinogenic mechanism of KDM4B in many tumors has not been deeply discussed and needs to be further studied, such as myeloma, lung cancer, leukemia, and classical Hodgkin lymphoma. In future, further studies on KDM4B in different tumors and tissues will provide more reliable evidence for whether KDM4B can be used as a tumor marker or even as a gene target for tumor therapy.

## 4 The Regulation of KDM4B Activity

### 4.1 Intracellular Regulators of KDM4B

Identified as endogenous regulatory factors of KDM4B, several microRNAs, such as miRNA-491-5P, have been found to inhibit KDM4B expression by binding to the 3′ UTR of KDM4B mRNA, thereby inhibiting the proliferation of breast cancer and gastric cancer cells (Zeng et al., 2015; Zhang et al., 2018). There are also several factors that promote the transcription of KDM4B under hypoxic conditions. HIF-1α recognizes and binds to the CCAAT sequence in the promoter sequence of the KDM4B gene, thereby inducing transcription of KDM4B (Beyer et al., 2008). During adipocyte differentiation, CCAAT/C/EBP β binds to the promoter of KDM4B and activates KDM4B transcription (Guo et al., 2012). In colorectal cancer cells, the transcription factor CREB directly binds to the appropriate region of the KDM4B promoter to activate KDM4B expression and induces radiation therapy resistance (Deng et al., 2018). DLX5 enhances the expression of KDM4B by binding to the promoter of KDM4B during osteoblast differentiation of dental tissue-derived mesenchymal stem cells (Yang et al., 2019). In human mesenchymal stem cells, the transcription factor TGF-β significantly increases KDM4B expression, thereby enhancing chondroblast differentiation ability (Lee et al., 2016).

**FIGURE 2:**
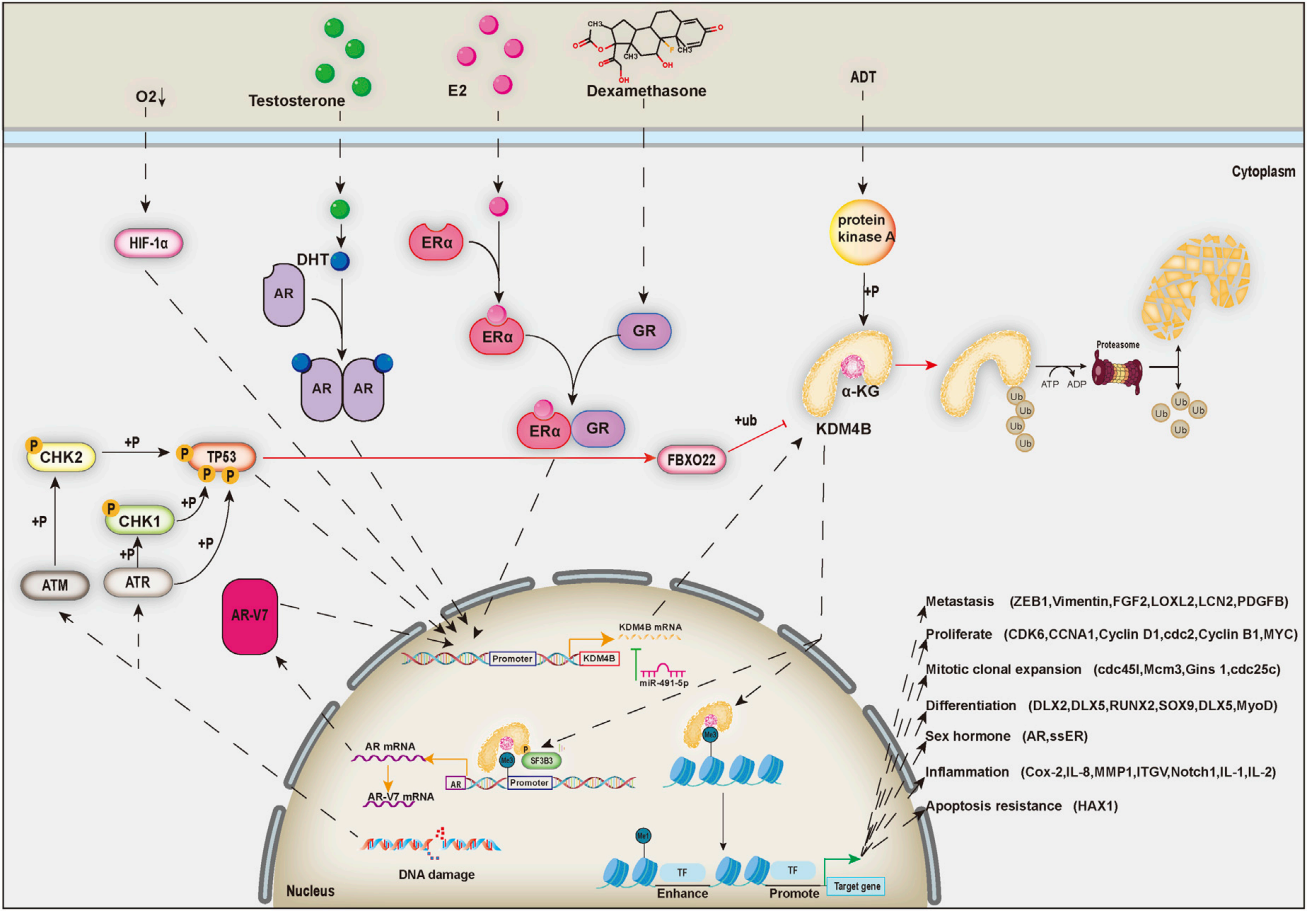
KDM4B plays an intermediate role in cell signal transduction.

In addition, the molecular chaperone heat shock protein 90 (Hsp90) can inhibit the ubiquitination of KDM4B by interacting with it (Ipenberg et al., 2013). Therefore, geldanamycin, a pharmacological inhibitor of Hsp90, can increase the ubiquitination level of KDM4B and thus indirectly decrease the KDM4B protein level (Ipenberg et al., 2013). Recent studies have found that mild stress-induced activation of TP53 can transcriptionally induce fbxo22, increase the ubiquitination level of KDM4B and directly degrade the KDM4B protein, while the phosphorylation of KDM4B by *p*-AKT1 can inhibit this process (Suzuki et al., 2021) (Figure 2).

### 4.2 Extracellular Regulators of KDM4B

#### 4.2.1 Regulation of KDM4B in a Hypoxic Environment

In tumors, hypoxia is an important extracellular regulator that leads to increased HIF-1α levels (Semenza, 2009). Several earlier reports have found that HIF-1α is associated with KDM4B upregulation under hypoxia (Beyer et al., 2008; Pollard et al., 2008). HIF-1α is stably expressed under hypoxia (Salminen et al., 2016)and could recognize and bind to the CCAAT sequence in the promoter sequence of the KDM4B gene, thus inducing increased transcription of KDM4B (Beyer et al., 2008). In breast cancer cells, HIF-1α upregulates the expression of KDM4B and further promotes the progression of breast cancer in association with ERα (Yang et al., 2010). In colorectal cancer cells, KDM4B expression is induced by HIF-1α, and KDM4B upregulates the expression of hypoxia-inducing genes such as carbonic anhydrase 9 (CA9) by decreasing H3K9me3 markers in the promoter (Fu et al., 2012). In gastric cancer cells, KDM4B binds to the promoter CCNA1 to result in CCNA1 upregulation under hypoxic conditions, thereby promoting cancer cell proliferation (Kim et al., 2012). In epithelial ovarian cancer cells, hypoxia leads to upregulation of the KDM4B gene, which reduces H3K9me markers in the promoters of transfer-related genes such as LOXL2, LCN2, and PDGFB, thereby increasing the expression of these genes and ultimately facilitating ovarian cancer cell migration and invasion (Wilson et al., 2017).

#### 4.2.2 Sex Hormones *and Regulation of KDM4B*

Sex hormones, mainly produced by sex organs, can act on sex hormone receptors extracellularly, thereby triggering a cascade of reactions that promote KDM4B expression (Duan et al., 2019b; Jones et al., 2019). Studies have shown that KDM4B is highly expressed in breast cancer cells with positive ERα expression (Yang et al., 2010). ERα is activated by binding to the estradiol 17-beta-estradiol (E2), which induces the expression of KDM4B (Kawazu et al., 2011; Shi et al., 2011). KDM4B is recruited to the ERα target to demethylate H3K9me3 markers and promote the transcription of ER responsive genes such as MYB, MYC, and CCND1 (Kawazu et al., 2011). Furthermore, KDM4B is also essential for ER transcription and is a key cascade signaling protein (Gaughan et al., 2013). In breast cancer cell lines, KDM4B demethylation of repressive H3K9me3 marks within upstream regulatory ER promoter regions permits binding of GATA-3 to drive ER expression (Gaughan et al., 2013). KDM4B has been found to be regulated by dihydrotestosterone (DHT) and AR, and they both bind to the KDM4B promoter region to promote the transcription of the KDM4B gene (Coffey et al., 2013). In addition, KDM4B not only participates in the transcription of AR downstream genes, but also inhibits the ubiquitination and degradation of AR (Coffey et al., 2013).

#### 4.2.3 Other Extracellular Regulators

Radiation therapy is a common cancer treatment and is generally used in 45–60% of cancer patients (Xiang K. et al., 2020). Radiation was a negative regulator of KDM4B expression and regulates global histone methylation of cancer cells (Kim et al., 2012). The radiation-mediated reduction in KDM4B inhibited CCNA1 expression under hypoxia condition, which severely impeded gastric cancer cell proliferation (Kim et al., 2012). Moreover, recent studies have also found that some metabolites produced by mutant isocitrate dehydrogenase-1 (IDH1) or-2 (IDH2), such as 2-hydroxyglutarate, succinate, and fumarate, can also significantly reduce the protein expression of KDM4B and inhibit DNA repair process (Sulkowski et al., 2020). In conclusion, regulation of histone demethylase KDM4B, which is a potential therapeutic target, might improve the efficacy of cancer treatment.

KDM4B is transcriptionally promoted by multiple transcription factors and repressed by miR-491-5p at the RNA level and ubiquitination of FBXO22 at the protein level. Ultimately, the main function of KDM4B is to switch H3K9me3 to H3K9me1 to turn on the expression of downstream genes via histone demethylation. In addition, when phosphorylated, KDM4B binds to the splicing factor SF3B3 to splice the newly synthesized mRNA of AR in the chromatin region.

## 5 KDM4 Inhibitors as Emerging Epigenetic Cancer Agents

### 5.1 1,5-Bis[(E)-2-(3,4-dichlorophenyl) ethenyl]-2,4-Dinitrobenzene (NSC636819)

1,5-Bis [(E)-2-(3,4-dichlorophenyl) ethenyl]-2,4-dinitrobenzene, NSC636819, was the first KDM4A/KDM4B inhibitor to be discovered and reported, with inhibition rates of 28% (KDM4A) and 35% (KDM4B) (Chu et al., 2014). This compound competitively binds to the active demethylase site in the JmjC domain of KDM4A and KDM4B, thus blocking their demethylation ability. In LNCaP cell lines, NSC636819 inhibits the histone demethylation of KDM4A and KDM4B as well as upregulates the H3K9me3 level, ultimately inhibiting the proliferation of prostate cancer cells (Chu et al., 2014). Interestingly, this compound has less effect on normal prostate cells than on prostate cancer cells when used at the same concentration, suggesting that it may have potential as a targeted agent for prostate cancer therapy (Chu et al., 2014). Currently, NSC636819 has no other use, and it has not been tested in animal or clinical trials.

### 5.2 B3 (NCGC00244536)

B3, NCGC00244536, was previously identified as a KDM4 inhibitor (Table 1). However, a recent study has shown that B3 exhibits a stronger inhibitory effect on KDM4B than on other KDM4 proteins. Therefore, NCGC00244536 is now considered as a KDM4B inhibitor (Duan et al., 2015). B3 directly binds to the demethylase catalytic site of the KDM4 protein, thereby inhibiting the activity of KDM4B (Duan et al., 2015). B3 inhibits the proliferation of various cancer cells, such as prostate, breast and cervical cancer cells, and it has little toxicity and few side effects in normal tissue cells (Duan et al., 2015). In a xenotransplantation model using PC3 cell-derived tumors in mice, B3 significantly inhibits tumor growth, and the mice show no significant toxicity or side effects (Duan et al., 2015). This result suggests that B3 has great potential as a gene-targeted drug to inhibit prostate cancer.

**TABLE 1:** Application of KDM4B related inhibitors in various diseases.

Inhibitors	Target	Disease type	References
1,5-bis [(E)-2-(3,4-dichlorophenyl) ethenyl]-2,4-dinitrobenzene	KDM4A/KDM4B	Prostate cancer	Chu et al. (2014)
B3	KDM4B	Prostate cancer	Duan et al. (2015)
QC6352 (2-OG analogue)	KDM4A-D	Breast cancer	Chen et al. (2017), Metzger et al. (2017)
CPX	KDM4B	Fungal infection, neuroblastoma	Subissi et al. (2010), Shen and Huang (2016), Yang et al. (2017)

### 5.3 2-Oxoglutarate (2-OG) Analogs

Fe (II) and 2-oxoglutarate (2-OG) oxygenases are necessary factors for histone demethylation in the JmjC domain of KDM4 family proteins (Hamada et al., 2010). 2-OG helps to add oxygen atoms to the methyl group to facilitate subsequent demethylation (Rose et al., 2008). Therefore, the catalytic activity of KDM4 family proteins is easily inhibited by 2-OG analogs (Laukka et al., 2018). 2-OG analogs can compete with 2-OG oxygenases to bind to the JmjC domain, but they do not have oxygenase activity (Hamada et al., 2010).

QC6352, a 2-OG analog developed and reported by Chen et al., in 2017, is an effective KDM4 family inhibitor obtained through continuous optimization of drug structure (Chen et al., 2017). Multiple experimental results have validated that the IC50 value of QC6352 for the inhibition of KDM4B activity is approximately 56 ± 6 nm (Chen et al., 2017; Metzger et al., 2017). The pyridine carboxylate core of QC6352 binds to the Jumonji C domain, forming a structure similar to that created when 2-OG binds and forms hydrogen bonds with Y132 and K206 of the KDM4 protein functional region, which prevents it from performing histone demethylation (Chen et al., 2017). Currently, QC6352 is mainly used to treat breast cancer. QC6352 has been found to block the proliferation of breast cancer stem cells and xenograft neoplasia, and it has been shown to have promising *in vivo* efficacy in a breast cancer patient-derived xenograft (PDX) (Chen et al., 2017). QC6352 blocks the proliferation of breast cancer stem cells and xenograft tumor formation (Metzger et al., 2017). In addition QC6352 can also inhibit the expression of epidermal growth factor receptor (EGFR) (Metzger et al., 2017), which may explain the decrease in tumor-initiating cell populations associated with chemotherapy resistance (Chen et al., 2017).

### 5.4 Ciclopirox

Ciclopirox (CPX), a member of the hydroxypyridinone class, has a high affinity for trivalent metal cations and has long been used as a common topical antifungal agent in clinical practice (Subissi et al., 2010). Further research on CPX has shown that it inhibits neuroblastoma, improves diabetes and its complications as well as prevents HIV infection. Interestingly, the functionality of CPX is related to its ability to chelate metal ions (Shen and Huang, 2016). Yang *et al.* found that CPX weakens the histone demethylase activity of KDM4B by inducing molecular interactions between nickel ions and the KDM4B protein, thus indirectly inhibiting the downstream MYC signaling pathway and mitochondrial oxidative phosphorylation in neuroblastoma, which inhibits the proliferation of neuroblastoma (Yang et al., 2017). Due to its low toxicity, CPX may have potential as a cancer-targeted drug that indirectly targets MYC (Table 1).

## 6 Conclusion

The KDM4B protein contains 1,096 amino acids and is mainly composed of four functional domains as follows: JmjN, JmjC, PHD, and Tudor. The JmjC domain is responsible for histone demethylation and plays a major role, but its function requires assistance from the JmjN domain (Shin and Janknecht, 2007b). The PHD and Tudor domains also help the protein target specific histone lysines, preventing KDM4B from functioning without regulation (Iwase et al., 2007). KDM4B is essentially a histone demethylase, and like most histone demethylases, it eliminates trimethylation of histone lysines, opens up chromatin, and enables the expression and translation of downstream genes (Fodor et al., 2006). The main carcinogenic effect of KDM4B is due to its ability to activate more oncogene signaling pathways after abnormal expression.

KDM4B and KDM4 family proteins play an important role in cell differentiation and organ development. KDM4B is critical for the maintenance of embryonic stem cells. KDM4B not only exerts histone demethylation ability to promote stem cell proliferation by interacting with Nanog at specific locations (Das et al., 2014), but also ensures the continuous feeding ability of trophoblast cells (Mak et al., 2021). Overexpression of KDM4B improves the survival rate of embryos *in vitro* (Antony et al., 2013). However, because KDM4B helps maintain an undifferentiated state (Wulansari et al., 2021), the overexpression of KDM4B may also be detrimental to the normal differentiation and development of stem cells. KDM4B can also promote the expression of the inflammation-related genes Notch1, IL-1, and IL-2, and excessive expression may cause adverse inflammatory responses (Das et al., 2013). Therefore, KDM4B is necessary for stem cell self-renewal and differentiation, but its level needs to be appropriate in mesenchymal stem cells. Because KDM4B can promote adipose differentiation or osteogenic differentiation as needed. Loss of KDM4B may lead to adverse conditions such as hyperlipidemia and osteoporosis (Ye et al., 2012; Cheng et al., 2018). KDM4B can also directly induce and activate the expression of sex hormone-related proteins, and there is a significant positive correlation between KDM4B and the expression of these proteins in the breast and prostate, indicating that KDM4B is an indispensable protein for sexual maturation (Shin and Janknecht, 2007a; Gaughan et al., 2013).

Because of its ability to promote gene expression, KDM4B is utilized as an intermediate binding protein by many oncoproteins in various types of cancers. Therefore, KDM4B is often considered as an oncogene in cancers (Berry and Janknecht, 2013). In cancer research, the KDM4B protein itself has not been reported to induce carcinogenesis through the generation of mutations. However, in cancer, the carcinogenic mechanism of KDM4B is due to an abnormal increase in its expression, resulting in activation of multiple oncogene signaling pathways by histone demethylation, such as binding with MYC, AR or ER to promote the expression of downstream genes (Coffey et al., 2013; Yang et al., 2015; West et al., 2016; Tang et al., 2020). In these reported cancers, the expression of KDM4B in cancer tissues is indeed higher than that in normal tissues, which indicates abnormal expression.

Currently, many inhibitors of KDM4B and the entire family of proteins are being tested and developed. Scientists suggest that KDM4B is an effective target and that blocking it could disrupt the interactions between cancer cell proteins (Stavropoulos and Hoelz, 2007). However, whether the inhibitors of KDM4B are effective and harmless to humans has not been reported. According to previous studies, KDM4B plays an irreplaceable role in the normal human body. Therefore, large-scale use of KDM4B inhibitors may inhibit the activity of KDM4B in normal cells, which may lead to adverse consequences, such as gonadal hypoplasia, osteoporosis and even obesity. Therefore, the use of KDM4B inhibitors requires reasonable assessment of the dosage and scope of action. As mentioned above, further exploring the mechanism of KDM4B in normal and tumor tissues can we provide more reliable evidence for whether KDM4B will be used as a tumor marker or even as a gene target for tumor therapy.
